# *catena*-Poly[[(1,10-phenanthroline-κ^2^ *N*,*N*′)copper(I)]-μ-thiocyanato-κ^2^ *N*:*S*-[(1,10-phenanthroline-κ^2^ *N*,*N*′)copper(I)]-μ-cyanido-κ^2^ *N*:*C*]

**DOI:** 10.1107/S1600536808037744

**Published:** 2008-11-20

**Authors:** Jun Zhao, Wen-Wen Dong, Dong-Sheng Li, Xi-Jun Ke

**Affiliations:** a College of Mechanical and Material Engineering, Functional Materials Research Institue, Three Gorges University, Yichang 443002, People’s Republic of China

## Abstract

In the title complex, [Cu_2_(CN)(NCS)(C_12_H_8_N_2_)_2_], which was synthesized under hydro­thermal conditions, both Cu^I^ atoms have a slightly distorted tetra­hedral geometry. They are coordinated by two N atoms of one 1,10-phenanthroline ligand, one bridging thio­cyanate anion and one bridging cyanide anion. In the crystal structure, infinite helical {Cu–CN–Cu–SCN}_*n*_ chains are formed along [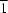
01].

## Related literature

For related literature, see: Cheng *et al.* (2006 ▶); Greig & Philp (2001 ▶); Luan *et al.* (2006 ▶); Piguet *et al.* (1997 ▶).
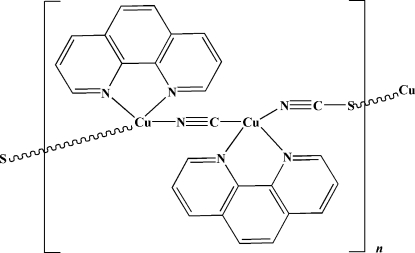


## Experimental

### 

#### Crystal data

[Cu_2_(CN)(NCS)(C_12_H_8_N_2_)_2_]
*M* *_r_* = 571.59
Monoclinic, 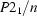

*a* = 13.046 (7) Å
*b* = 13.470 (7) Å
*c* = 13.538 (7) Å
β = 90.044 (9)°
*V* = 2379 (2) Å^3^
*Z* = 4
Mo *K*α radiation
μ = 1.90 mm^−1^
*T* = 293 (2) K
0.30 × 0.15 × 0.12 mm

#### Data collection

Bruker SMART CCD diffractometer
Absorption correction: multi-scan *SADABS* (Sheldrick, 1996 ▶) *T*_min_ = 0.599, *T*_max_ = 0.804
15496 measured reflections
4959 independent reflections
3662 reflections with *I* > 2σ(*I*)
*R*_int_ = 0.081

#### Refinement

*R*[*F* ^2^ > 2σ(*F* ^2^)] = 0.061
*wR*(*F* ^2^) = 0.164
*S* = 1.00
4959 reflections
316 parameters
H-atom parameters constrained
Δρ_max_ = 0.47 e Å^−3^
Δρ_min_ = −0.57 e Å^−3^

### 

Data collection: *SMART* (Bruker, 1997 ▶); cell refinement: *SAINT* (Bruker, 1997 ▶); data reduction: *SAINT*; program(s) used to solve structure: *SHELXS97* (Sheldrick, 2008 ▶); program(s) used to refine structure: *SHELXL97* (Sheldrick, 2008 ▶); molecular graphics: *SHELXTL* (Sheldrick, 2008 ▶); software used to prepare material for publication: *SHELXTL*.

## Supplementary Material

Crystal structure: contains datablocks I, New_Global_Publ_Block. DOI: 10.1107/S1600536808037744/bt2813sup1.cif

Structure factors: contains datablocks I. DOI: 10.1107/S1600536808037744/bt2813Isup2.hkl

Additional supplementary materials:  crystallographic information; 3D view; checkCIF report

## Figures and Tables

**Fig. 1.**
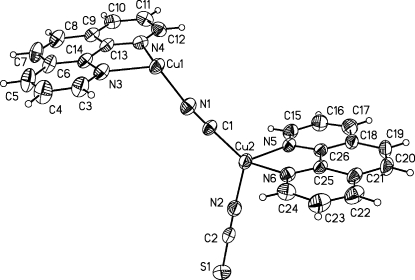
The structure of the title compound, with displacement ellipsoids for the non-hydrogen atoms drawn at the 30% probability level.

**Fig. 2.**
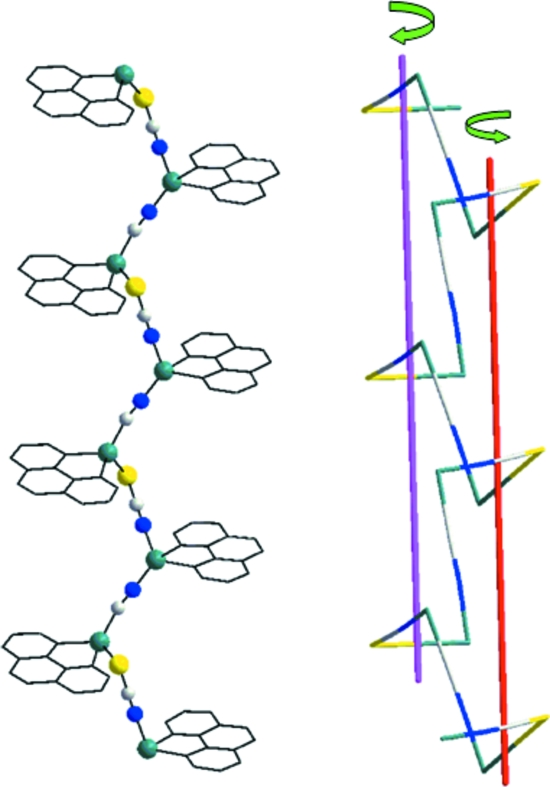
Left: presentation of the location of the copper centres of coordination polymer; Right: View of the meso-helical arrangement.

## supplementary crystallographic information

### Comment

Self-assembly processes that lead to helical structures are common throughout biology and chemistry (Luan *et al.*, 2006; Piguet *et al.*, 2005). Protein α-helices and the DNA double helix are well known biological examples which have inspired the work of synthetic chemists aiming to create chemical analogs of these complex structures (Greig *et al.*, 2001). However, there is a little known about *meso*-helical self-assembling systems within this very active field of helical structure research in supramolecular chemistry (Cheng *et al.*, 2006).

The crystal structure of the title complex contains two 1,10-Phen ligands, one CuSCN and one CuCN co-existing in the asymmetric unit, as illustrated in Fig. 1. The coordianation geometry of the four-coordinated Cu(1) is slightly distorted tetrahedral with two N donors of the chelating 1,10-Phen and another N donor [N(1)] of the CN^-^ occupying the basal sites and a S donor of SCN^-^ occupying the vertex site. The Cu(2) also has a slightly distorted tetrahedral geometry and is coordinated by two N atoms of one 1,10-Phen ligand, one bridging thiocyanate anion N atom [N(2)] and one bridging cyanide C atom [C(1)]. It is noteworthy that the Cu(I)atoms are linked by CN^-^ and SCN^-^ anions into infinite helical {CuCN-CuSCN}*_n_* chains along a 2_1_ screw axis, furthermore, the Cu_2_(CN)(SCN) chains run around and cross two parallel axes forming *meso*-helices as showed in Fig. 2.

### Experimental

All chemicals were of reagent grade quality obtained from commercial sources and used without further purification. A mixture of CuSCN (0.60 mmol, 0.07 g), NaCN (1 mmol, 0.05 g),1,10-Phen (0.40 mmol, 0.07 g) and water (10 ml) in a 25 ml Teflon-lined stainless steel reactor was heated from 298 to 453 K in 2 h and maintained at 453 K for 72 h. After the mixture wascooled to 298 K, red crystals of the title compound were obtained  (yield 43%).

### Refinement

All H atoms were positioned geometrically (C—H = 0.93 Å) and allowed to ride on their parent atoms, with *U*_iso_(H) values equal to 1.2*U*_eq_(C).

### Crystal data

**Table:**

[Cu_2_(CN)(NCS)(C_12_H_8_N_2_)_2_]	*F*_000_ = 1152
*M**_r_* = 571.59	*D*_x_ = 1.596 Mg m^−^^3^
Monoclinic, *P*2_1_/*n*	Mo *K*α radiation λ = 0.71073 Å
Hall symbol: -P 2yn	Cell parameters from 2103 reflections
*a* = 13.046 (7) Å	θ = 2.2–27.5º
*b* = 13.470 (7) Å	µ = 1.90 mm^−^^1^
*c* = 13.538 (7) Å	*T* = 293 (2) K
β = 90.044 (9)º	Prism, red
*V* = 2379 (2) Å^3^	0.30 × 0.15 × 0.12 mm
*Z* = 4	

### Data collection

**Table:**

Bruker SMART CCD diffractometer	4959 independent reflections
Radiation source: fine-focus sealed tube	3662 reflections with *I* > 2σ(*I*)
Monochromator: graphite	*R*_int_ = 0.081
Detector resolution: 13.6612 pixels mm^-1^	θ_max_ = 27.5º
*T* = 293(2) K	θ_min_ = 2.2º
CCD Profile fitting scans	*h* = −15→16
Absorption correction: multi-scanSADABS (Sheldrick, 1996)	*k* = −17→15
*T*_min_ = 0.599, *T*_max_ = 0.804	*l* = −17→17
15496 measured reflections	

### Refinement

**Table:**

Refinement on *F*^2^	Secondary atom site location: difference Fourier map
Least-squares matrix: full	Hydrogen site location: inferred from neighbouring sites
*R*[*F*^2^ > 2σ(*F*^2^)] = 0.061	H-atom parameters constrained
*wR*(*F*^2^) = 0.164	*w* = 1/[σ^2^(*F*_o_^2^) + (0.0669*P*)^2^ + 0.4979*P*] where *P* = (*F*_o_^2^ + 2*F*_c_^2^)/3
*S* = 1.00	(Δ/σ)_max_ = 0.001
4959 reflections	Δρ_max_ = 0.47 e Å^−^^3^
316 parameters	Δρ_min_ = −0.57 e Å^−^^3^
Primary atom site location: structure-invariant direct methods	Extinction correction: none

### Special details

**Table:**

Geometry. All e.s.d.'s (except the e.s.d. in the dihedral angle between two l.s. planes) are estimated using the full covariance matrix. The cell e.s.d.'s are taken into account individually in the estimation of e.s.d.'s in distances, angles and torsion angles; correlations between e.s.d.'s in cell parameters are only used when they are defined by crystal symmetry. An approximate (isotropic) treatment of cell e.s.d.'s is used for estimating e.s.d.'s involving l.s. planes.
Refinement. Refinement of *F*^2^ against ALL reflections. The weighted *R*-factor *wR* and goodness of fit *S* are based on *F*^2^, conventional *R*-factors *R* are based on *F*, with *F* set to zero for negative *F*^2^. The threshold expression of *F*^2^ > σ(*F*^2^) is used only for calculating *R*-factors(gt) *etc*. and is not relevant to the choice of reflections for refinement. *R*-factors based on *F*^2^ are statistically about twice as large as those based on *F*, and *R*- factors based on ALL data will be even larger.

### Fractional atomic coordinates and isotropic or equivalent isotropic displacement parameters (Å^2^)

**Table:**

	*x*	*y*	*z*	*U*_iso_*/*U*_eq_	
Cu1	0.08441 (4)	0.73813 (3)	0.04132 (4)	0.06012 (19)	
Cu2	0.45032 (3)	0.76203 (3)	−0.04863 (3)	0.05589 (18)	
C1	0.3089 (3)	0.7520 (2)	−0.0233 (3)	0.0502 (8)	
N2	0.5019 (2)	0.8164 (2)	−0.1747 (2)	0.0623 (7)	
N3	−0.0405 (2)	0.73312 (19)	−0.0551 (2)	0.0557 (7)	
N4	0.0024 (2)	0.86301 (19)	0.0898 (2)	0.0524 (6)	
N5	0.54334 (18)	0.8232 (2)	0.06500 (19)	0.0501 (6)	
N6	0.5591 (2)	0.64667 (19)	−0.02813 (19)	0.0515 (6)	
N1	0.2232 (2)	0.74724 (19)	−0.0059 (2)	0.0619 (8)	
C2	0.5338 (2)	0.8478 (2)	−0.2473 (2)	0.0505 (7)	
C3	−0.0636 (3)	0.6686 (3)	−0.1262 (3)	0.0712 (10)	
H3A	−0.0226	0.6125	−0.1325	0.085*	
C4	−0.1433 (4)	0.6793 (3)	−0.1905 (4)	0.0901 (14)	
H4A	−0.1549	0.6324	−0.2397	0.108*	
C5	−0.2064 (4)	0.7608 (3)	−0.1815 (4)	0.0854 (15)	
H5A	−0.2610	0.7697	−0.2248	0.103*	
C6	−0.1878 (2)	0.8295 (2)	−0.1071 (3)	0.0609 (9)	
C7	−0.2500 (3)	0.9176 (3)	−0.0924 (3)	0.0709 (10)	
H7A	−0.3060	0.9296	−0.1333	0.085*	
C8	−0.2269 (3)	0.9819 (3)	−0.0200 (3)	0.0662 (10)	
H8A	−0.2675	1.0380	−0.0119	0.079*	
C9	−0.1417 (2)	0.9670 (2)	0.0451 (3)	0.0523 (7)	
C10	−0.1156 (3)	1.0327 (2)	0.1207 (3)	0.0597 (9)	
H10A	−0.1545	1.0896	0.1311	0.072*	
C11	−0.0333 (3)	1.0133 (3)	0.1790 (3)	0.0682 (10)	
H11A	−0.0155	1.0563	0.2299	0.082*	
C12	0.0254 (3)	0.9263 (3)	0.1610 (2)	0.0625 (9)	
H12A	0.0820	0.9135	0.2008	0.075*	
C13	−0.0798 (2)	0.8824 (2)	0.0330 (2)	0.0473 (7)	
C14	−0.1038 (2)	0.8128 (2)	−0.0458 (2)	0.0502 (7)	
C15	0.5371 (3)	0.9102 (3)	0.1122 (3)	0.0639 (9)	
H15A	0.4849	0.9536	0.0943	0.077*	
C16	0.6044 (3)	0.9392 (3)	0.1865 (3)	0.0747 (10)	
H16A	0.5968	1.0008	0.2167	0.090*	
C17	0.6812 (3)	0.8776 (3)	0.2149 (3)	0.0733 (10)	
H17A	0.7253	0.8954	0.2659	0.088*	
C18	0.6929 (3)	0.7856 (3)	0.1653 (3)	0.0559 (8)	
C19	0.7759 (3)	0.7185 (3)	0.1877 (3)	0.0692 (10)	
H19A	0.8219	0.7332	0.2382	0.083*	
C20	0.7865 (3)	0.6345 (3)	0.1351 (3)	0.0696 (10)	
H20A	0.8424	0.5937	0.1478	0.084*	
C21	0.7157 (2)	0.6058 (2)	0.0611 (3)	0.0581 (8)	
C22	0.7226 (3)	0.5174 (3)	0.0063 (3)	0.0743 (12)	
H22A	0.7770	0.4740	0.0168	0.089*	
C23	0.6491 (3)	0.4955 (3)	−0.0622 (3)	0.0783 (12)	
H23A	0.6530	0.4370	−0.0985	0.094*	
C24	0.5680 (3)	0.5616 (3)	−0.0775 (3)	0.0641 (9)	
H24A	0.5182	0.5455	−0.1240	0.077*	
C25	0.6313 (2)	0.6691 (2)	0.0399 (2)	0.0471 (7)	
C26	0.6224 (3)	0.7617 (2)	0.0929 (2)	0.0468 (7)	
S1	0.58145 (8)	0.89734 (6)	−0.34839 (7)	0.0644 (3)	

### Atomic displacement parameters (Å^2^)

**Table:**

	*U*^11^	*U*^22^	*U*^33^	*U*^12^	*U*^13^	*U*^23^
Cu1	0.0442 (3)	0.0592 (3)	0.0770 (4)	0.01178 (17)	0.0058 (2)	0.00483 (19)
Cu2	0.0407 (3)	0.0623 (3)	0.0646 (3)	0.00543 (17)	−0.0032 (2)	0.00538 (18)
C1	0.0387 (17)	0.0418 (15)	0.070 (2)	0.0058 (12)	0.0049 (15)	0.0057 (13)
N2	0.0498 (15)	0.0711 (19)	0.0659 (18)	−0.0020 (14)	−0.0025 (13)	0.0112 (15)
N3	0.0468 (16)	0.0429 (14)	0.077 (2)	0.0043 (11)	0.0045 (15)	−0.0008 (12)
N4	0.0538 (14)	0.0452 (13)	0.0582 (15)	0.0046 (12)	0.0092 (12)	0.0053 (12)
N5	0.0421 (13)	0.0504 (14)	0.0577 (15)	0.0087 (11)	0.0024 (11)	−0.0010 (12)
N6	0.0503 (14)	0.0457 (14)	0.0584 (15)	0.0028 (11)	0.0049 (12)	0.0001 (12)
N1	0.0516 (19)	0.0502 (16)	0.084 (2)	0.0083 (12)	0.0027 (16)	0.0065 (14)
C2	0.0389 (14)	0.0447 (16)	0.068 (2)	0.0002 (13)	−0.0035 (14)	−0.0008 (14)
C3	0.061 (2)	0.056 (2)	0.097 (3)	0.0088 (17)	−0.003 (2)	−0.023 (2)
C4	0.084 (3)	0.067 (3)	0.119 (4)	0.008 (2)	−0.017 (3)	−0.036 (3)
C5	0.060 (3)	0.069 (3)	0.127 (4)	0.0007 (19)	−0.027 (3)	−0.022 (2)
C6	0.0396 (15)	0.0487 (17)	0.094 (3)	−0.0007 (14)	−0.0049 (16)	−0.0069 (17)
C7	0.0495 (18)	0.0507 (19)	0.113 (3)	0.0065 (16)	−0.0176 (19)	−0.005 (2)
C8	0.0518 (19)	0.0413 (17)	0.106 (3)	0.0098 (15)	−0.0004 (19)	0.0024 (18)
C9	0.0471 (16)	0.0394 (15)	0.071 (2)	0.0027 (13)	0.0081 (15)	0.0049 (14)
C10	0.067 (2)	0.0431 (17)	0.069 (2)	0.0091 (15)	0.0118 (18)	0.0027 (15)
C11	0.089 (3)	0.056 (2)	0.060 (2)	0.006 (2)	0.004 (2)	0.0001 (16)
C12	0.071 (2)	0.060 (2)	0.0563 (19)	0.0092 (18)	−0.0005 (16)	0.0008 (16)
C13	0.0384 (14)	0.0383 (15)	0.0653 (18)	0.0014 (12)	0.0091 (13)	0.0044 (13)
C14	0.0395 (14)	0.0389 (15)	0.072 (2)	0.0000 (12)	0.0074 (14)	0.0024 (14)
C15	0.0590 (19)	0.060 (2)	0.072 (2)	0.0170 (17)	0.0047 (17)	−0.0110 (17)
C16	0.078 (2)	0.074 (2)	0.072 (2)	0.011 (2)	−0.001 (2)	−0.023 (2)
C17	0.071 (2)	0.085 (3)	0.065 (2)	−0.006 (2)	−0.0035 (18)	−0.016 (2)
C18	0.0430 (17)	0.066 (2)	0.0590 (19)	−0.0033 (16)	−0.0031 (14)	0.0115 (17)
C19	0.049 (2)	0.086 (3)	0.072 (2)	0.003 (2)	−0.0150 (17)	0.017 (2)
C20	0.0497 (18)	0.073 (2)	0.086 (3)	0.0160 (18)	−0.0044 (18)	0.030 (2)
C21	0.0468 (17)	0.0500 (18)	0.077 (2)	0.0118 (14)	0.0091 (16)	0.0149 (16)
C22	0.072 (3)	0.052 (2)	0.099 (3)	0.0215 (19)	0.020 (2)	0.013 (2)
C23	0.092 (3)	0.047 (2)	0.097 (3)	0.012 (2)	0.021 (3)	−0.005 (2)
C24	0.075 (2)	0.0519 (18)	0.065 (2)	0.0020 (17)	0.0054 (17)	−0.0055 (16)
C25	0.0397 (14)	0.0446 (15)	0.0570 (17)	0.0076 (12)	0.0079 (13)	0.0080 (13)
C26	0.0429 (17)	0.0476 (16)	0.0500 (17)	0.0051 (12)	0.0054 (14)	0.0045 (12)
S1	0.0725 (6)	0.0491 (5)	0.0717 (6)	−0.0137 (4)	0.0150 (5)	−0.0022 (4)

### Geometric parameters (Å, °)

**Table:**

Cu1—N1	1.924 (3)	C8—H8A	0.9300
Cu1—N3	2.089 (3)	C9—C10	1.395 (5)
Cu1—N4	2.099 (3)	C9—C13	1.406 (4)
Cu1—S1^i^	2.3581 (13)	C10—C11	1.358 (5)
Cu2—C1	1.882 (3)	C10—H10A	0.9300
Cu2—N2	1.976 (3)	C11—C12	1.420 (5)
Cu2—N6	2.123 (3)	C11—H11A	0.9300
Cu2—N5	2.125 (3)	C12—H12A	0.9300
C1—N1	1.145 (5)	C13—C14	1.454 (5)
N2—C2	1.148 (4)	C15—C16	1.391 (5)
N3—C3	1.331 (5)	C15—H15A	0.9300
N3—C14	1.360 (4)	C16—C17	1.356 (6)
N4—C12	1.322 (4)	C16—H16A	0.9300
N4—C13	1.345 (4)	C17—C18	1.417 (5)
N5—C15	1.336 (4)	C17—H17A	0.9300
N5—C26	1.376 (4)	C18—C26	1.382 (5)
N6—C24	1.331 (4)	C18—C19	1.443 (5)
N6—C25	1.351 (4)	C19—C20	1.343 (6)
C2—S1	1.645 (3)	C19—H19A	0.9300
C3—C4	1.363 (6)	C20—C21	1.416 (5)
C3—H3A	0.9300	C20—H20A	0.9300
C4—C5	1.377 (6)	C21—C22	1.406 (5)
C4—H4A	0.9300	C21—C25	1.421 (4)
C5—C6	1.390 (6)	C22—C23	1.366 (6)
C5—H5A	0.9300	C22—H22A	0.9300
C6—C14	1.392 (5)	C23—C24	1.399 (5)
C6—C7	1.452 (5)	C23—H23A	0.9300
C7—C8	1.341 (5)	C24—H24A	0.9300
C7—H7A	0.9300	C25—C26	1.444 (4)
C8—C9	1.433 (5)	S1—Cu1^ii^	2.3581 (13)
			
N1—Cu1—N3	121.90 (14)	C9—C10—H10A	120.1
N1—Cu1—N4	122.18 (11)	C10—C11—C12	119.1 (3)
N3—Cu1—N4	79.83 (11)	C10—C11—H11A	120.5
N1—Cu1—S1^i^	105.99 (9)	C12—C11—H11A	120.5
N3—Cu1—S1^i^	110.96 (8)	N4—C12—C11	122.4 (3)
N4—Cu1—S1^i^	114.43 (8)	N4—C12—H12A	118.8
C1—Cu2—N2	121.25 (14)	C11—C12—H12A	118.8
C1—Cu2—N6	125.39 (12)	N4—C13—C9	123.2 (3)
N2—Cu2—N6	98.99 (11)	N4—C13—C14	117.7 (3)
C1—Cu2—N5	117.10 (13)	C9—C13—C14	119.0 (3)
N2—Cu2—N5	106.65 (12)	N3—C14—C6	123.4 (3)
N6—Cu2—N5	78.93 (11)	N3—C14—C13	116.6 (3)
N1—C1—Cu2	178.3 (4)	C6—C14—C13	120.1 (3)
C2—N2—Cu2	178.7 (3)	N5—C15—C16	123.6 (3)
C3—N3—C14	116.5 (3)	N5—C15—H15A	118.2
C3—N3—Cu1	130.5 (2)	C16—C15—H15A	118.2
C14—N3—Cu1	112.9 (2)	C17—C16—C15	119.9 (4)
C12—N4—C13	118.2 (3)	C17—C16—H16A	120.1
C12—N4—Cu1	129.0 (2)	C15—C16—H16A	120.1
C13—N4—Cu1	112.6 (2)	C16—C17—C18	118.8 (4)
C15—N5—C26	116.2 (3)	C16—C17—H17A	120.6
C15—N5—Cu2	130.6 (2)	C18—C17—H17A	120.6
C26—N5—Cu2	113.1 (2)	C26—C18—C17	117.9 (3)
C24—N6—C25	118.3 (3)	C26—C18—C19	120.1 (4)
C24—N6—Cu2	128.5 (3)	C17—C18—C19	122.0 (4)
C25—N6—Cu2	113.0 (2)	C20—C19—C18	119.6 (4)
C1—N1—Cu1	172.5 (4)	C20—C19—H19A	120.2
N2—C2—S1	177.3 (3)	C18—C19—H19A	120.2
N3—C3—C4	124.4 (4)	C19—C20—C21	122.5 (3)
N3—C3—H3A	117.8	C19—C20—H20A	118.7
C4—C3—H3A	117.8	C21—C20—H20A	118.7
C3—C4—C5	118.9 (4)	C22—C21—C20	124.2 (3)
C3—C4—H4A	120.5	C22—C21—C25	116.8 (4)
C5—C4—H4A	120.5	C20—C21—C25	118.9 (3)
C4—C5—C6	119.4 (4)	C23—C22—C21	119.7 (3)
C4—C5—H5A	120.3	C23—C22—H22A	120.1
C6—C5—H5A	120.3	C21—C22—H22A	120.1
C14—C6—C5	117.5 (3)	C22—C23—C24	119.5 (4)
C14—C6—C7	119.4 (3)	C22—C23—H23A	120.2
C5—C6—C7	123.1 (4)	C24—C23—H23A	120.2
C8—C7—C6	120.1 (3)	N6—C24—C23	122.7 (4)
C8—C7—H7A	119.9	N6—C24—H24A	118.7
C6—C7—H7A	119.9	C23—C24—H24A	118.7
C7—C8—C9	122.2 (3)	N6—C25—C21	122.9 (3)
C7—C8—H8A	118.9	N6—C25—C26	118.4 (3)
C9—C8—H8A	118.9	C21—C25—C26	118.7 (3)
C10—C9—C13	117.4 (3)	N5—C26—C18	123.6 (3)
C10—C9—C8	123.4 (3)	N5—C26—C25	116.4 (3)
C13—C9—C8	119.1 (3)	C18—C26—C25	120.0 (3)
C11—C10—C9	119.7 (3)	C2—S1—Cu1^ii^	102.69 (12)
C11—C10—H10A	120.1		
			
N2—Cu2—C1—N1	−122 (11)	Cu1—N4—C13—C14	4.5 (3)
N6—Cu2—C1—N1	107 (11)	C10—C9—C13—N4	−0.5 (4)
N5—Cu2—C1—N1	11 (11)	C8—C9—C13—N4	179.1 (3)
C1—Cu2—N2—C2	178 (100)	C10—C9—C13—C14	−179.2 (3)
N6—Cu2—N2—C2	−41 (13)	C8—C9—C13—C14	0.3 (4)
N5—Cu2—N2—C2	40 (13)	C3—N3—C14—C6	−1.2 (5)
N1—Cu1—N3—C3	59.4 (4)	Cu1—N3—C14—C6	175.1 (3)
N4—Cu1—N3—C3	−178.9 (3)	C3—N3—C14—C13	178.9 (3)
S1^i^—Cu1—N3—C3	−66.5 (3)	Cu1—N3—C14—C13	−4.8 (4)
N1—Cu1—N3—C14	−116.3 (2)	C5—C6—C14—N3	−0.3 (5)
N4—Cu1—N3—C14	5.5 (2)	C7—C6—C14—N3	−179.0 (3)
S1^i^—Cu1—N3—C14	117.9 (2)	C5—C6—C14—C13	179.6 (4)
N1—Cu1—N4—C12	−58.1 (3)	C7—C6—C14—C13	0.9 (5)
N3—Cu1—N4—C12	−179.6 (3)	N4—C13—C14—N3	0.2 (4)
S1^i^—Cu1—N4—C12	71.9 (3)	C9—C13—C14—N3	179.0 (3)
N1—Cu1—N4—C13	116.1 (2)	N4—C13—C14—C6	−179.7 (3)
N3—Cu1—N4—C13	−5.3 (2)	C9—C13—C14—C6	−0.9 (4)
S1^i^—Cu1—N4—C13	−113.87 (19)	C26—N5—C15—C16	−0.8 (5)
C1—Cu2—N5—C15	−55.7 (3)	Cu2—N5—C15—C16	179.4 (3)
N2—Cu2—N5—C15	83.8 (3)	N5—C15—C16—C17	−0.3 (6)
N6—Cu2—N5—C15	−180.0 (3)	C15—C16—C17—C18	2.0 (6)
C1—Cu2—N5—C26	124.4 (2)	C16—C17—C18—C26	−2.6 (5)
N2—Cu2—N5—C26	−96.0 (2)	C16—C17—C18—C19	176.6 (4)
N6—Cu2—N5—C26	0.2 (2)	C26—C18—C19—C20	2.5 (5)
C1—Cu2—N6—C24	67.3 (3)	C17—C18—C19—C20	−176.7 (4)
N2—Cu2—N6—C24	−71.8 (3)	C18—C19—C20—C21	−3.5 (6)
N5—Cu2—N6—C24	−177.2 (3)	C19—C20—C21—C22	−178.3 (4)
C1—Cu2—N6—C25	−117.6 (2)	C19—C20—C21—C25	1.4 (5)
N2—Cu2—N6—C25	103.2 (2)	C20—C21—C22—C23	178.7 (3)
N5—Cu2—N6—C25	−2.1 (2)	C25—C21—C22—C23	−0.9 (5)
Cu2—C1—N1—Cu1	−39 (13)	C21—C22—C23—C24	0.3 (6)
N3—Cu1—N1—C1	−177 (2)	C25—N6—C24—C23	−0.6 (5)
N4—Cu1—N1—C1	85 (2)	Cu2—N6—C24—C23	174.2 (3)
S1^i^—Cu1—N1—C1	−49 (2)	C22—C23—C24—N6	0.5 (6)
Cu2—N2—C2—S1	−66 (17)	C24—N6—C25—C21	−0.2 (4)
C14—N3—C3—C4	2.1 (6)	Cu2—N6—C25—C21	−175.7 (2)
Cu1—N3—C3—C4	−173.4 (3)	C24—N6—C25—C26	179.4 (3)
N3—C3—C4—C5	−1.3 (8)	Cu2—N6—C25—C26	3.9 (3)
C3—C4—C5—C6	−0.3 (8)	C22—C21—C25—N6	0.9 (5)
C4—C5—C6—C14	1.1 (7)	C20—C21—C25—N6	−178.7 (3)
C4—C5—C6—C7	179.7 (4)	C22—C21—C25—C26	−178.7 (3)
C14—C6—C7—C8	−0.4 (6)	C20—C21—C25—C26	1.7 (4)
C5—C6—C7—C8	−179.0 (4)	C15—N5—C26—C18	0.1 (5)
C6—C7—C8—C9	−0.2 (6)	Cu2—N5—C26—C18	180.0 (2)
C7—C8—C9—C10	179.7 (4)	C15—N5—C26—C25	−178.1 (3)
C7—C8—C9—C13	0.2 (5)	Cu2—N5—C26—C25	1.8 (3)
C13—C9—C10—C11	−0.1 (5)	C17—C18—C26—N5	1.6 (5)
C8—C9—C10—C11	−179.7 (3)	C19—C18—C26—N5	−177.7 (3)
C9—C10—C11—C12	0.6 (5)	C17—C18—C26—C25	179.7 (3)
C13—N4—C12—C11	−0.2 (5)	C19—C18—C26—C25	0.5 (5)
Cu1—N4—C12—C11	173.8 (2)	N6—C25—C26—N5	−3.8 (4)
C10—C11—C12—N4	−0.4 (5)	C21—C25—C26—N5	175.8 (3)
C12—N4—C13—C9	0.6 (4)	N6—C25—C26—C18	177.9 (3)
Cu1—N4—C13—C9	−174.3 (2)	C21—C25—C26—C18	−2.5 (4)
C12—N4—C13—C14	179.4 (3)	N2—C2—S1—Cu1^ii^	163 (6)

Symmetry codes: (i) *x*−1/2, −*y*+3/2, *z*+1/2; (ii) *x*+1/2, −*y*+3/2, *z*−1/2.
